# Association between fatty acid metabolism gene mutations and Mycobacterium tuberculosis transmission revealed by whole genome sequencing

**DOI:** 10.1186/s12866-023-03072-9

**Published:** 2023-12-01

**Authors:** Yameng Li, Xianglong Kong, Yifan Li, Ningning Tao, Tingting Wang, Yingying Li, Yawei Hou, Xuehan Zhu, Qilin Han, Yuzhen Zhang, Qiqi An, Yao Liu, Huaichen Li

**Affiliations:** 1 Deartment of Chinese Medicine Integrated with Western Medicine, College of Traditional Chinese Medicine, Shandong University of Traditional Chinese Medicine, 16369 Jingshi Road, Lixia District, Jinan, 250355 Shandong People’s Republic of China; 2 Artificial Intelligence Institute, Qilu University of Technology (Shandong Academy of Sciences), Jinan, 250011 Shandong People’s Republic of China; 3 Department of Respiratory and Critical Care Medicine, The Third Affiliated Hospital of Shandong First Medical University (Affiliated Hospital of Shandong Academy of Medical Sciences), Jinan, 250031 Shandong People’s Republic of China; 4 Department of Respiratory and Critical Care Medicine, Shandong Provincial Hospital, Shandong University, Shandong Provincial Hospital Affiliated to Shandong First Medical University, Jingwuweiqi Road, Huaiyin District, Jinan, 250021 Shandong People’s Republic of China; 5 Shandong First Medical University & Shandong Academy of Medical Sciences, Jinan, 250117 Shandong People’s Republic of China

**Keywords:** Fatty acid metabolism gene mutations, Tuberculosis, Whole genome sequencing, Transmission

## Abstract

**Background:**

Fatty acid metabolism greatly promotes the virulence and pathogenicity of *Mycobacterium tuberculosis* (*M.tb*). However, the regulatory mechanism of fatty acid metabolism in *M.tb* remains to be elucidated, and limited evidence about the effects of gene mutations in fatty acid metabolism on the transmission of *M.tb* was reported.

**Results:**

Overall, a total of 3193 *M.tb* isolates were included in the study, of which 1596 (50%) were genomic clustered isolates. Most of the tuberculosis isolates belonged to lineage2(*n* = 2744,85.93%), followed by lineage4(*n* = 439,13.75%) and lineage3(*n* = 10,0.31%).Regression results showed that the mutations of gca (136,605, 317G > C, Arg106Pro; OR, 22.144; 95% CI, 2.591-189.272), ogt(1,477,346, 286G > C ,Gly96Arg; OR, 3.893; 95%CI, 1.432–10.583), and rpsA (1,834,776, 1235 C > T, Ala412Val; OR, 3.674; 95% CI, 1.217–11.091) were significantly associated with clustering; mutations in gca and rpsA were also significantly associated with clustering of lineage2. Mutation in arsA(3,001,498, 885 C > G, Thr295Thr; OR, 6.278; 95% CI, 2.508–15.711) was significantly associated with cross-regional clusters. We also found that 20 mutation sites were positively correlated with cluster size, while 11 fatty acid mutation sites were negatively correlated with cluster size.

**Conclusion:**

Our research results suggested that mutations in genes related to fatty acid metabolism were related to the transmission of *M.tb*. This research could help in the future control of the transmission of *M.tb*.

**Supplementary Information:**

The online version contains supplementary material available at 10.1186/s12866-023-03072-9.

## Background

Tuberculosis (TB) is a highly contagious infectious disease caused by *Mycobacterium tuberculosis(M.tb)* that primarily affects the lungs and spreads through the respiratory tract [1, 2]. It is estimated that 25% of the global population is infected with *M.tb*. Despite the large global efforts at curbing the spread of *M.tb* complex strains, 10.6 million new patients develop TB every year [3–5]. China still has the second-highest number of TB infections globally [6]. Therefore, having a thorough and comprehensive understanding of the transmission mechanisms of *M.tb* is of great significance in the prevention and treatment of TB.

The metabolism of fatty acids is critical to the survival of *M.tb* within the host. *M.tb* utilizes diverse lipids as major carbon and energy source during infection. Fatty acids are degraded via beta-oxidation to generate reduced power and energy [7–9]. At the same time, fatty acids play a crucial role in the composition of the cell wall of *M.tb* [10]. Furthermore, it is worth noting that *M.tb* utilizes fatty acids to produce essential metabolic intermediates closely related to its virulence [11]. *M.tb* is capable of incorporating fatty acids into phospholipids or utilizing them as a source of carbon for energy storage through their conversion into triglycerides. This conversion process has been linked to the promotion of drug resistance in *M.tb* [12, 13]. In response to hypoxia, *M.tb* within macrophages loaded with lipids undergoes a process of accumulating neutral lipids, which results in the loss of acid resistance and the development of antibiotic resistance [14]. Some virulence genes can facilitate the spread of *M.tb*. Fatty acid metabolism plays a significant role in enhancing the virulence and pathogenicity of *M.tb*. Nonetheless, the exact regulatory mechanism of fatty acid metabolism in *M.tb* is still unclear, and there is limited research on how mutations in fatty acid metabolism genes affect the transmission of *M.tb*. Therefore, further investigation is necessary to gain a better understanding of these aspects.

Whole genome sequencing (WGS) is a reliable tool for studying *M.tb* transmission. In this study, WGS was used to study the influence of fatty acid metabolism gene mutations on the transmission of *M.tb* in China. Specifically, the genomic cluster was used to represent the transmission of *M.tb* [15].

## Results

### Sample description

**Fig. 1:**
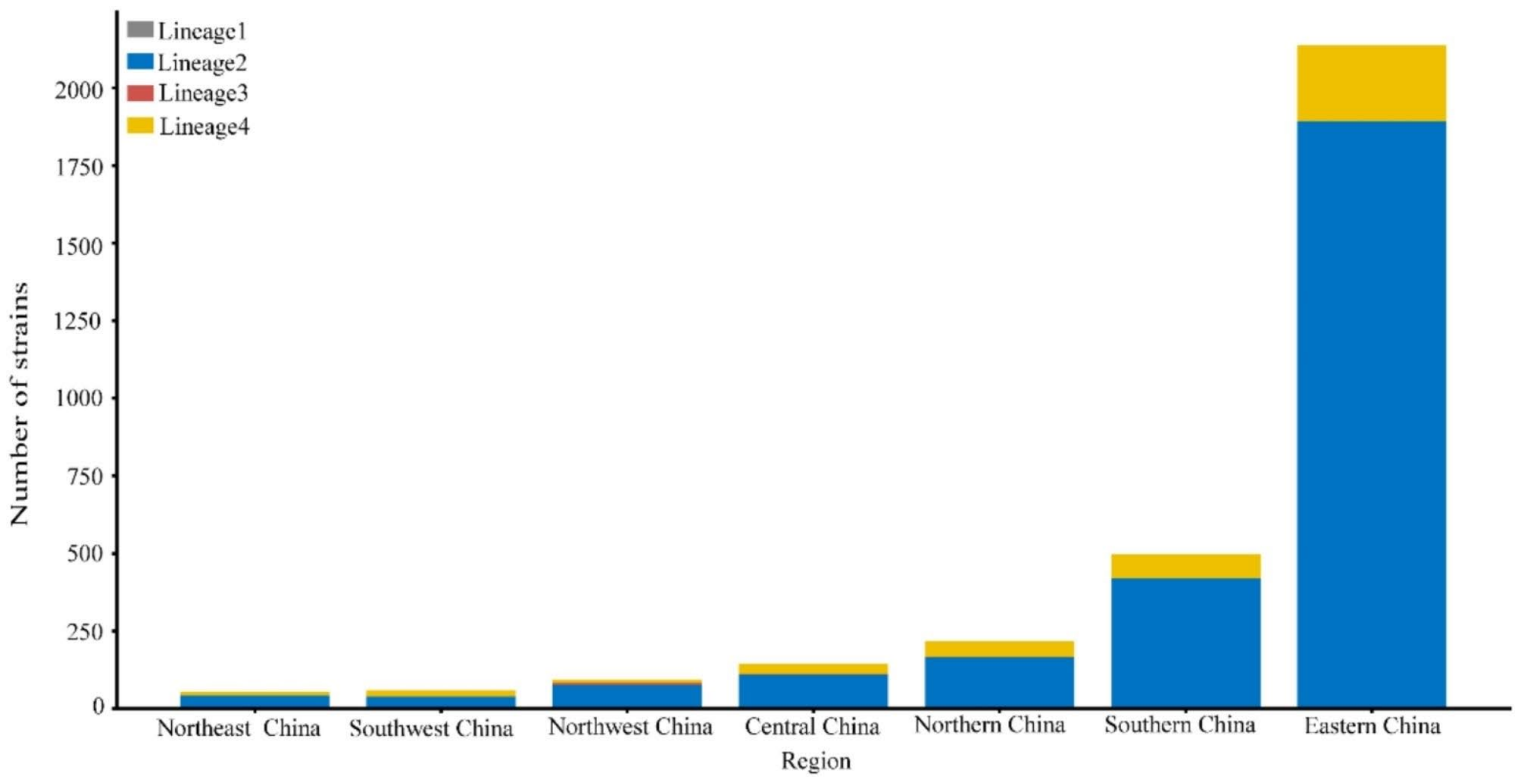
Distribution of 3197 isolates of *Mycobacterium tuberculosis* in seven natural geographical regions of China

**Fig. 2:**
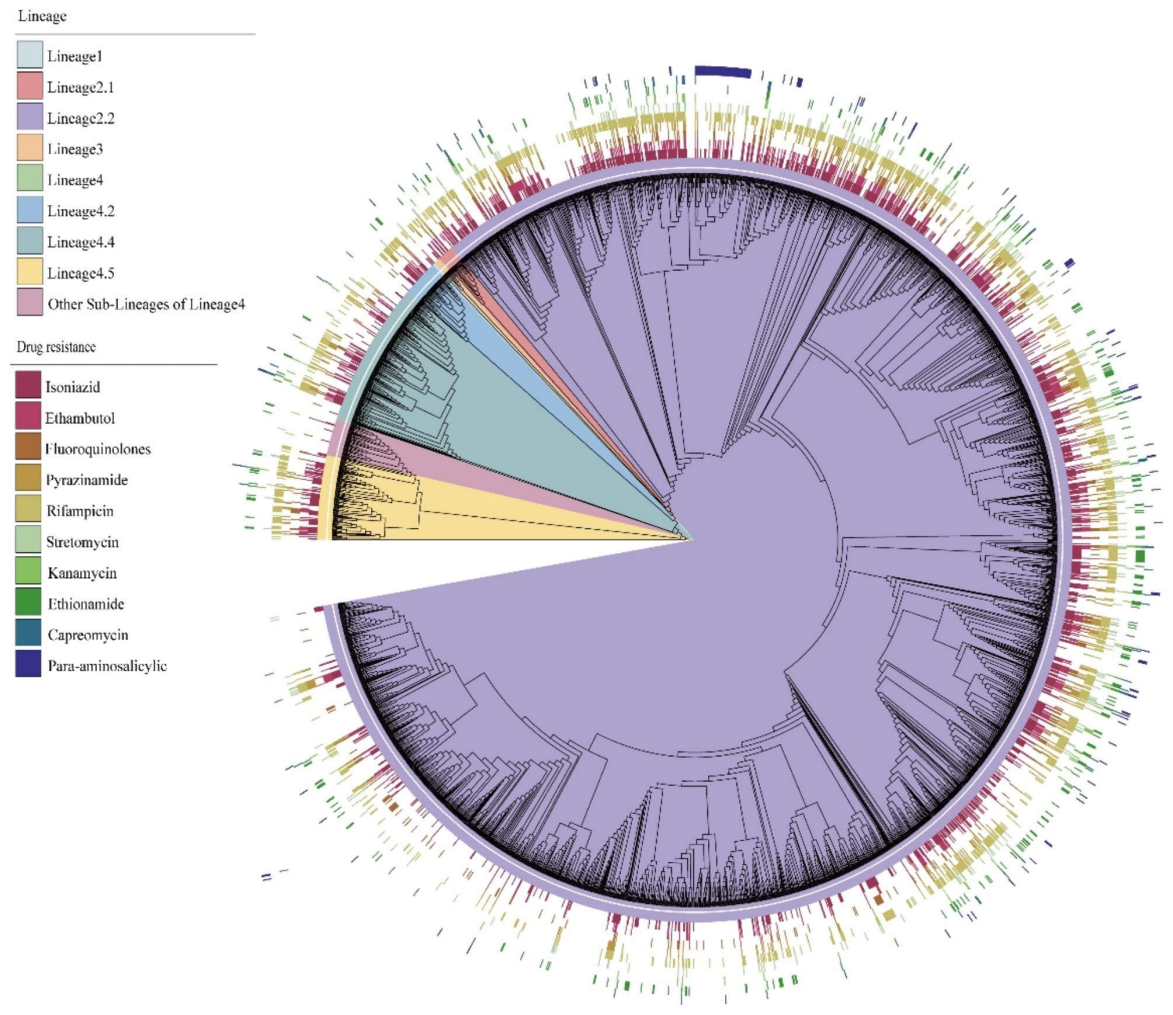
Phylogenetic tree for the 3197 *Mycobacterium tuberculosis* isolates from China

The *M.tb* isolates were classified according to the seven geographical regions of China. The vast majority of M.tb isolates from Eastern China (66.8%), Southern China (15.4%) and Central China (4.5%), as shown in Fig. 1. The analysis revealed that the majority of *M.tb* isolates belonged to lineage2 (*n* = 2744, 85.93%), followed by lineage4 (*n* = 439, 13.75%), and a smaller number of isolates belonged to lineage3 (*n* = 10, 0.31%). Most of the isolates belonged to sub-lineage2.2, while there were fewer isolates belonging to sub-lineage4.4 and sub-lineage4.5. The *M.tb* isolates were clustered into 499 groups, with sizes ranging from 2 to 108 isolates. Of these clusters, those containing 2 isolates of *M.tb* were defined as small clusters, those containing 3–5 isolates were defined as medium clusters, and those containing 6 or more isolates were defined as large clusters. There were 86 cross-regional clusters, ranging in size from 2 to 6 regions, as shown in Table 1. The phylogenetic tree of *M.tb* isolates was constructed as described in Fig. 2.

**Table 1:** Characteristics of *Mycobacterium tuberculosis* in China

Characteristic	Number (%)
**Lineage**	
Lineage2	2744(85.93)
Lineage3	10(0.31)
Lineage4	439(13.75)
**Sub-lineage**	
Lineage2.1	21(0.77)
Lineage2.2	2723(85.55)
Lineage4.2	49(1.54)
Lineage4.4	197(6.19)
Lineage4.5	184(5.78)
Other sub-lineage4	11(0.35)
**Isolates**	
Non-clusters	1597(50.00)
Clusters	1596(50.00)
**Clusters by size**	
2	606(38.00)
3 ~ 5	542(34.00)
6 or more	448(28.10)
**Region**	
Non-cross-regional	1189(74.36)
Cross-regional	407(25.64)

### The effect of mutations in fatty acid metabolism genes on clustering

After excluding positions with a mutation frequency lower than 0.01, we analyzed 73 mutation positions. During the comparison between clustered and non-clustered isolates, we observed significant differences (*P* < 0.05) for 43 mutation positions in fatty acid metabolism genes in the univariate analysis, as detailed in Supplement Table 1. Following univariate analysis, 73 mutation positions variables were selected for multivariate regression. To correct for possible confounding factors, we used the lineage and geographical location of *M.tb* as covariates in addition to the 73 mutation positions in fatty acid metabolism genes. Finally, five mutation positions of fatty acid metabolism genes with significant influence on clustering were determined (P < 0.05), as shown in Table 2. Among these, three mutation positions of fatty acid metabolism genes were identified as risk factors for clustering. The mutations were gca (136,605, 317G > C, Arg106Pro; OR, 22.144; 95% CI, 2.591-189.272), ogt(1,477,346, 286G > C ,Gly96Arg; OR, 3.893; 95%CI, 1.432–10.583), and rpsA (1,834,776, 1235 C > T, Ala412Val; OR, 3.674; 95% CI, 1.217–11.091), respectively.

**Table 2:** Analysis of the effect of fatty acid metabolism gene mutations on clustering

Gene	Position	Type	Reference	Variant	*P* Value	OR (95% CI)
ppiA	12,555	snp	C	T	1.000	
fadD34	37,305	snp	C	G	0.672	1.733(0.136–22.106)
fadD34	37,334	snp	A	T	0.037	0.168(0.032–0.898)
fadD34	37,553	ins	T	TTCATGACTC GGCTCGGCCCAC	0.858	1.068(0.52–2.191)
fadD34	37,971	snp	G	C	0.999	
fadD34	38,199	snp	C	T	0.818	1.379(0.09-21.158)
gca	136,605	snp	G	C	0.005	22.144(2.591-189.272)
lipC	262,829	snp	C	T	1.000	0.000
clpB	460,413	snp	C	T	1.000	0.000
fgd1	491,556	snp	G	A	0.282	1.704(0.646–4.498)
fgd1	491,742	snp	T	C	0.999	0.000
pepC	893,733	snp	T	G	0.029	0.171(0.035–0.831)
pepC	893,895	snp	C	T	1.000	
far	951,702	snp	C	T	0.019	0.298(0.109–0.818)
fadB	957,117	snp	T	C	1.000	
ercc3	958,607	snp	G	A	0.407	1.336(0.673–2.654)
ercc3	959,167	snp	C	T	0.999	
fadH	1,306,259	snp	A	G	0.708	0.631(0.057–7.037)
fadH	1,306,322	snp	C	G	0.805	1.155(0.368–3.618)
fadH	1,306,796	snp	G	A	0.439	1.928(0.366–10.172)
fadH	1,307,598	snp	C	G	0.728	1.967(0.043–89.207)
ogt	1,477,346	snp	C	G	0.008	3.893(1.432–10.583)
ogt	1,477,522	snp	C	A	0.365	0.296(0.021–4.135)
ogt	1,477,596	snp	C	T	1.000	
lipI	1,576,481	snp	T	G	0.999	
lipI	1,576,527	snp	G	T	0.899	1.113(0.214–5.774)
tkt	1,630,148	snp	A	C	1.000	
inhA	1,674,210	snp	A	C	0.001	0.288(0.142–0.584)
fadD11	1,754,459	snp	G	A	1.000	
lgt	1,814,428	snp	G	A	0.999	0.000
rpsA	1,834,177	snp	A	C	0.999	0.000
rpsA	1,834,776	snp	C	T	0.021	3.674(1.217–11.091)
tlyA	1,917,972	snp	A	G	0.999	0.000
lipJ	2,147,022	snp	A	C	0.732	1.313(0.276–6.256)
helZ	2,361,030	snp	G	A	0.999	0.000
helZ	2,361,311	snp	C	T	1.000	
helZ	2,361,604	snp	C	G	0.243	0.286(0.035–2.337)
helZ	2,362,041	snp	C	A	0.999	
fadD15	2,448,458	snp	C	T	0.999	
fadD15	2,449,629	snp	G	A	0.935	1.034(0.461–2.321)
dlaT	2,482,888	snp	G	A	1.000	
acpS	2,839,689	snp	C	T	0.067	0.737(0.532–1.021)
fas	2,841,022	snp	A	G	0.929	0.893(0.075–10.667)
fas	2,847,281	snp	A	G	0.286	4.655(0.277–78.322)
relA	2,908,252	snp	G	A	0.797	1.136(0.431–2.994)
arsA	3,001,498	snp	C	G	0.004	0.459(0.269–0.781)
arsA	3,001,785	snp	G	A	0.119	0.574(0.286–1.154)
mtr	3,165,636	snp	G	A	0.501	0.578(0.117–2.852)
tesA	3,242,617	snp	C	T	0.791	1.119(0.486–2.577)
gatB	3,367,765	snp	G	A	1.000	0.000
cstA	3,428,183	snp	G	A	0.390	0.859(0.607–1.216)
cstA	3,428,917	snp	C	A	0.999	0.000
agpS	3,476,350	snp	G	A	0.881	1.236(0.077–19.928)
nudC	3,571,828	snp	G	C	0.999	0.000
sdhD	3,704,686	snp	T	C	0.999	
sdhD	3,704,770	snp	A	G	0.152	6.16(0.511–74.19)
nagA	3,719,723	snp	A	C	0.576	0.633(0.127–3.145)
lipF	3,906,311	snp	G	A	0.815	1.266(0.176–9.081)
acs	4,108,495	snp	G	A	0.532	1.192(0.687–2.066)
acs	4,109,342	snp	G	A	0.566	0.897(0.62–1.299)
crp	4,116,610	snp	G	A	0.259	2.577(0.499–13.321)
crp	4,116,773	snp	T	C	0.999	0.000
pcnA	4,392,373	snp	G	A	0.964	1.018(0.461–2.248)

OR, Odds ratio; CI, confidence interval

-means there is no result in statistical software or the result was too large and nonsense

### Effects of mutations in fatty acid metabolism genes on clustering of lineage2

Positions with mutation in genes involved in fatty acid metabolism in lineage2 frequency less than 0.01 were removed. We analyzed 55 mutation positions, and 18 fatty acid metabolism gene mutation positions showed statistically significant differences between clustered and non-clustered isolates (*P* < 0.05), as detailed in Supplementary Table 2. Following univariate analysis, 55 mutation positions of fatty acid metabolism genes were analyzed by multivariate regression. In order to correct the possible confounding factors, we used the geographical location of *M.tb* as a covariate in addition to the 55 mutation positions in fatty acid metabolism genes. The results showed that mutations in six fatty acid metabolism gene positions were significantly associated with the clustered isolates of lineage2 (P < 0.05), see Table 3. Among these three mutation positions were identified as risk factors for clustering, including ogt (1,477,346, 286G > C, Gly96Arg; OR, 3.952; 95% CI, 1.453–10.749), rpsA (1,834,776, 1235 C > T, Ala412Val; OR,3.636; 95% CI,1.204–10.982), and gca (136,605, 317G > C, Arg106Pro; OR, 22.789; 95% CI, 2.669-194.569).

**Table 3:** Analysis of the effect of fatty acid metabolism gene mutations on clustering of lineage 2

Gene	Position	Type	Reference	Variant	*P* Value	OR (95% CI)
fadD34	37,305	snp	C	G	0.685	1.68(0.137–20.594)
fadD34	37,334	snp	A	T	0.039	0.172(0.033–0.914)
fadD34	37,971	snp	G	C	0.999	
fadD34	38,199	snp	C	T	0.849	1.302(0.086–19.663)
gca	136,605	snp	G	C	0.004	22.789(2.669-194.569)
lipC	262,829	snp	C	T	1.000	0.000
clpB	460,413	snp	C	T	1.000	0.000
fgd1	491,556	snp	G	A	0.294	1.682(0.637–4.441)
fgd1	491,742	snp	T	C	0.999	0.000
pepC	893,733	snp	T	G	0.034	0.172(0.034–0.875)
far	951,702	snp	C	T	0.018	0.296(0.108–0.812)
ercc3	958,607	snp	G	A	0.394	1.347(0.68–2.668)
fadH	1,306,259	snp	A	G	0.719	0.643(0.058–7.15)
fadH	1,307,598	snp	C	G	0.999	0.000
ogt	1,477,346	snp	C	G	0.007	3.952(1.453–10.749)
ogt	1,477,522	snp	C	A	0.379	0.309(0.023–4.223)
ogt	1,477,596	snp	C	T	1.000	
lipI	1,576,481	snp	T	G	0.998	
lipI	1,576,527	snp	G	T	0.939	1.066(0.206–5.518)
inhA	1,674,210	snp	A	C	0.001	0.296(0.146–0.599)
fadD11	1,754,459	snp	G	A	1.000	
lgt	1,814,428	snp	G	A	0.999	0.000
rpsA	1,834,177	snp	A	C	0.998	0.000
rpsA	1,834,776	snp	C	T	0.022	3.636(1.204–10.982)
tlyA	1,917,972	snp	A	G	0.999	0.000
lipJ	2,147,022	snp	A	C	0.876	1.139(0.222–5.856)
helZ	2,361,604	snp	C	G	0.999	0.000
helZ	2,362,041	snp	C	A	0.999	
fadD15	2,449,629	snp	G	A	0.952	1.025(0.458–2.293)
acpS	2,839,689	snp	C	T	0.091	0.754(0.544–1.046)
fas	2,841,022	snp	A	G	0.777	1.468(0.103–20.877)
fas	2,847,281	snp	A	G	0.293	4.527(0.272–75.447)
relA	2,908,252	snp	G	A	0.736	1.181(0.449–3.107)
arsA	3,001,498	snp	C	G	0.003	0.447(0.262–0.764)
arsA	3,001,785	snp	G	A	0.142	0.593(0.295–1.192)
mtr	3,165,636	snp	G	A	0.491	0.57(0.115–2.824)
tesA	3,242,617	snp	C	T	0.800	1.113(0.485–2.556)
cstA	3,428,183	snp	G	A	0.399	0.861(0.608–1.219)
cstA	3,428,917	snp	C	A	0.999	0.000
agpS	3,476,350	snp	G	A	0.921	1.15(0.072–18.234)
nudC	3,571,828	snp	G	C	0.998	0.000
sdhD	3,704,770	snp	A	G	0.135	6.658(0.554–79.965)
lipF	3,906,311	snp	G	A	0.806	1.28(0.179–9.147)
acs	4,108,495	snp	G	A	0.531	1.192(0.688–2.063)
acs	4,109,342	snp	G	A	0.601	0.906(0.626–1.311)
crp	4,116,610	snp	G	A	0.239	2.687(0.518–13.952)

OR, Odds ratio; CI, confidence interval

-means there is no result in statistical software or the result was too large and nonsense

### Effects of mutations in fatty acid metabolism genes on clustering of lineage4

The analysis focused on 33 fatty acid metabolism gene mutation positions in lineage4, which were selected by excluding those with frequencies lower than 0.01. In the comparison between clustered and non-clustered isolates of lineage4, the difference in the mutation of two fatty acid metabolism gene positions was statistically significant (*P* < 0.05). Results can be found in Supplementary Table 3. Following univariate analysis, 33 fatty acid metabolism gene mutation positions were included in a multivariate regression analysis. However, we included the geographical location of *M.tb* as a covariate in our analysis in order to control for possible confounding effects. The results showed that there was no risk factor for the clustered isolates of lineage4, see Table 4.

**Table 4:** Analysis of the effect of fatty acid metabolism gene mutations on clustering of lineage 4

Gene	Position	Type	Reference	Variant	*P* Value	OR (95% CI)
fadD34	37,553	ins	T	TTCATGACTCG GCTCGGCCCAC	0.859	1.07(0.504–2.273)
pepC	893,733	snp	T	G	0.999	0.000
pepC	893,895	snp	C	T	1.000	
fadB	957,117	snp	T	C	1.000	
ercc3	959,167	snp	C	T	0.584	1.426(0.4-5.078)
fadH	1,306,322	snp	C	G	0.94	1.048(0.31–3.539)
fadH	1,306,796	snp	G	A	0.488	1.812(0.337–9.734)
fadH	1,307,598	snp	C	G	1.000	
tkt	1,630,148	snp	A	C	0.999	0.000
lipJ	2,147,022	snp	A	C	1.000	
helZ	2,361,311	snp	C	T	1.000	
helZ	2,361,604	snp	C	G	0.781	0.67(0.04-11.224)
helZ	2,362,041	snp	C	A	0.999	
dlaT	2,482,888	snp	G	A	1.000	
acpS	2,839,689	snp	C	T	1.000	0.000
gatB	3,367,765	snp	G	A	1.000	0.000
cstA	3,428,917	snp	C	A	0.999	0.000
sdhD	3,704,686	snp	T	C	0.999	
nagA	3,719,723	snp	A	C	0.562	0.62(0.123–3.13)
crp	4,116,773	snp	T	C	0.999	0.000
pcnA	4,392,373	snp	G	A	0.923	0.96(0.415–2.222)

OR, Odds ratio; CI, confidence interval

-means there is no result in statistical software or the result was too large and nonsense

### The effect of mutations in fatty acid metabolism genes on the cross-regional transmission of ***M.tb***

After screening out the positions with clustering mutation frequency less than 0.01, 61 mutation positions of fatty acid metabolism genes were analyzed. In comparison between the cross-regional and non-cross-regional clusters, 26 fatty acid metabolism gene mutation positions showed significant differences (*P* < 0.05), as detailed in Supplementary Table 4. Following univariate analysis, 61 mutation positions were included in multiple regression analysis, and we also included the lineage as covariate to correct for potential confounding factors. The results showed that five mutation positions of fatty acid metabolism genes had a significant influence on regional factors (*P* < 0.05), see Table 5. Among these, mutation position of arsA(3,001,498) was identified as cross-regional risk factors (885 C > G, Thr295Thr; OR, 6.278; 95% CI, 2.508–15.711). Notably, the arsA was synonymous mutations.

**Table 5:** Analysis of the influence of fatty acid metabolism gene mutations on cross-regional

Gene	Position	Type	Reference	Variant	*P* Value	OR (95% CI)
ppiA	12,555	snp	C	T	1.000	
fadD34	37,305	snp	C	G	1.000	
fadD34	37,334	snp	A	T	0.999	
fadD34	37,553	ins	T	TTCATGACTCGG CTCGGCCCAC	0.5	1.689(0.368–7.743)
fadD34	37,971	snp	G	C	0.443	1.518(0.523–4.406)
fadD34	38,199	snp	C	T	0.999	0.000
gca	136,605	snp	G	C	1.000	1.000
lipC	262,829	snp	C	T	0.087	3.095(0.848–11.293)
clpB	460,413	snp	C	T	0.998	0.000
fgd1	491,556	snp	G	A	0.999	
fgd1	491,742	snp	T	C	1.000	
pepC	893,733	snp	T	G	0.272	0.481(0.13–1.776)
far	951,702	snp	C	T	0.997	
fadB	957,117	snp	T	C	1.000	
ercc3	958,607	snp	G	A	0.948	1.032(0.398–2.676)
ercc3	959,167	snp	C	T	1.000	1.000
fadH	1,306,259	snp	A	G	0.999	
fadH	1,306,322	snp	C	G	1.000	1.000
fadH	1,307,598	snp	C	G	1.000	
ogt	1,477,346	snp	C	G	0.015	0.136(0.028–0.674)
ogt	1,477,522	snp	C	A	0.999	
lipI	1,576,481	snp	T	G	1.000	
lipI	1,576,527	snp	G	T	0.999	0.000
inhA	1,674,210	snp	A	C	0.999	0.000
rpsA	1,834,177	snp	A	C	1.000	0.000
rpsA	1,834,776	snp	C	T	0.998	0.000
tlyA	1,917,972	snp	A	G	0.999	
lipJ	2,147,022	snp	A	C	0.999	
helZ	2,361,311	snp	C	T	1.000	0.000
helZ	2,361,604	snp	C	G	0.503	2.581(0.161–41.285)
fadD15	2,449,629	snp	G	A	0.999	0.000
acpS	2,839,689	snp	C	T	0.019	0.467(0.247–0.88)
fas	2,841,022	snp	A	G	0.716	0.654(0.066–6.436)
fas	2,847,281	snp	A	G	1.000	
relA	2,908,252	snp	G	A	5.175e-5	0.013(0.002–0.108)
arsA	3,001,498	snp	C	G	8.681e-5	6.278(2.508–15.711)
mtr	3,165,636	snp	G	A	0.998	0.000
tesA	3,242,617	snp	C	T	0.001	0.059(0.011–0.302)
gatB	3,367,765	snp	G	A	1.000	1.000
cstA	3,428,183	snp	G	A	0.221	0.691(0.383–1.248)
cstA	3,428,917	snp	C	A	0.999	
agpS	3,476,350	snp	G	A	0.999	0.000
nudC	3,571,828	snp	G	C	1.000	
sdhD	3,704,770	snp	A	G	0.999	
nagA	3,719,723	snp	A	C	0.112	0.259(0.049–1.372)
lipF	3,906,311	snp	G	A	0.999	0.000
acs	4,108,495	snp	G	A	0.073	0.38(0.132–1.094)
acs	4,109,342	snp	G	A	0.147	0.619(0.323–1.184)
crp	4,116,610	snp	G	A	0.998	
crp	4,116,773	snp	T	C	0.999	
pcnA	4,392,373	snp	G	A	0.998	

OR, Odds ratio; CI, confidence interval

-means there is no result in statistical software or the result was too large and nonsense

### Effects of mutations in fatty acid metabolism genes on cluster size of ***M.tb***

**Fig. 3:**
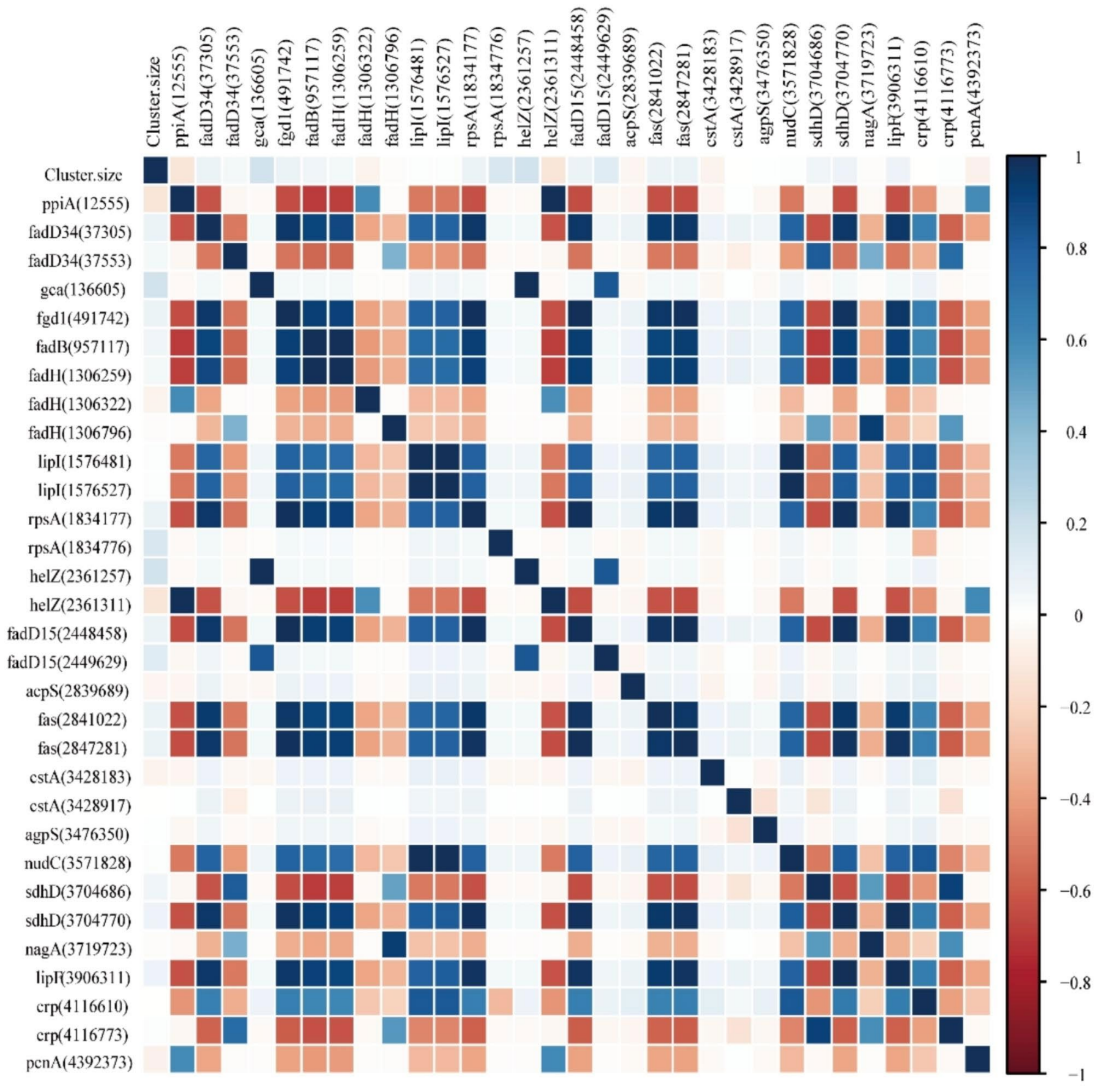
Correlation analysis of fatty acid metabolism gene mutation positions and clusters

A total of 61 mutation positions of fatty acid metabolism genes were analyzed. The results showed that 31 mutation positions were significantly associated with cluster size (*P* < 0.05). Among these, 20 mutation positions were found to be positively related to cluster size. Notably, seven of these mutation positions were synonymous, including fgd1 (491,742, 960T > C, Phe320Phe), fadB (957,117, 825T > C, Asp275Asp), fadH (1,306,259, 1968T > C, Ala656Ala), rpsA (1,834,177, 636 A > C, Arg212Arg), fadD15 (2,449,629, 1470G > A, Gln490Gln), fas (2,847,281, 2052T > C, Asp684Asp), and agpS (3,476,350, 612 C > T, Ser204Ser). For further details refer to Fig. 3.

## Discussion

Fatty acid metabolism plays a crucial role in the growth of *M.tb*. To investigate the impact mutations of fatty acid metabolism gene mutations on the spread of TB in China, we analyzed 3107 isolates of *M.tb* and 83 fatty acid metabolism genes. In China, most of the *M.tb* isolates belonged to lineage2 (Beijing lineage), followed by lineage 4 (European lineage), and lineage3 (South Asia lineage).Most of the clustered isolates (*n* = 1463,91.67%) also belonged to lineage2, which indicated that the main isolates of transmission belonged to lineage2 in China.

Based on our findings, we observed a missense mutation (317G > C, Arg106Pro) at position 136,605 of gca (Rv0112), and another missense mutation (1235 C > T, Ala412Val) at position 1,834,776 of rpsA (Rv1630). These mutations have been associated with increased risk of transmission of *M.tb*, particularly within lineage 2, and are also correlated with cluster size. Although some functions of gca remain unclear, they may be associated with the transport of the *M.tb* cell membrane and the synthesis of the cell wall, both of which play critical roles in the pathogenesis of TB. Further research is needed to fully understand the mechanism by which this mutation promotes transmission. RpsA (Rv1630) is the largest 30 S protein in the ribosome and plays a crucial role in translation. Mutations or deletions of rpsA can have a significant impact on the growth and metabolism of M.tb [16–18]. A missense mutation (c.1235 C > T p.Ala412Val) has been identified at position 1,834,776 of rpsA. This mutation promotes the spread of TB isolates and lineage2 isolates and is associated with cluster size. Interestingly, both the Beijing isolate of M.tb and multidrug-resistant isolates exhibit two non-synonymous single nucleotide polymorphisms in the ogt gene [19–21]. The researchers hypothesized that these mutations in ogt (Rv1316c) may contribute to the successful global distribution of these isolates, which is consistent with our findings. Our results revealed a missense mutation (286 g > C, Gly 96 arg) at position 1,477,346 of the ogt gene. The ogt gene encodes an enzyme called N-acetylglucosamine (O-GlcNAc) transferase, which is a glycosyltransferase responsible for catalyzing the addition of O-GlcNAc modification onto specific serine or threonine residues of proteins. O-GlcNAc transferase may play a role in regulating *M.tb* growth, adaptability, and pathogenicity by modifying and affecting key *M.tb* proteins. This missense mutation potentially promotes the transmission of *M.tb* isolates, including lineage 2 isolates, and could have implications fo*r M.tb* metabolism, cell wall synthesis, drug resistance, and other characteristics [22, 23]. In our study, we did not find any mutations in fatty acid metabolism genes that had an impact on the transmission of lineage 4. This could be partially attributed to the fact that our sample size may have been insufficient to capture rare lineage 4 strains or related mutations, and a larger sample set might provide more accurate results.

A synonymous mutation at position 3,001,498 of arsA (Rv2684) (885 C > G, Thr295Thr) has been found to affect the transmission of isolates across different regions. The expression of arsA allows *M.tb* to adapt to different environments within the host’s body. Specifically, arsA helps the bacterium to evade the host immune response [2, 24].

In addition, our results confirmed that both synonymous and non-synonymous mutations can affect the transmission of *M.tb*, indicating that synonymous mutations in fatty acid metabolism of *M.tb* are not all neutral mutations, which is consistent with the result that synonymous mutations in yeast genes studied by Xukang Shen are mostly strong non-neutral mutations [25].

## Conclusion

The results of this study suggest that mutations in fatty acid metabolism genes may increase the transmission risk of *M.tb*, which highlights the need for further investigation into the effects of these mutations on *M.tb* control and dissemination. These findings provide valuable insights into the therapy of TB.

## Method

### Sample collection

A total of 1550 *M.tb* culture-positive cases were collected from two medical institutions from 2011 to 2018 in China: Shandong Public Health Clinical Research Center (SPHCC) and Weifang Respiratory Clinical Hospital (WRCH). All samples were collected anonymously and informed consent was not required. Our research was approved by the Ethics Committee of Shandong Provincial Hospital, which is affiliated with Shandong First Medical University.

### DNA extraction and sequencing

Genomic DNA from 1447 isolates was extracted with Cetyltrimethylammonium Bromide (CTAB) and underwent quality control (QC). The Illumina HiSeq 4000 system was used to sequence the genomes [26], and the sequence data were deposited in the National Center for Biotechnology Information (NCBI) under BioProject PRJNA1002108. In addition, 1755 isolates of *M.tb* from 23 provinces, 4 municipalities, and 5 autonomous regions in China were included in this study [27–34]. See Supplementary Tables 5–6 for the sample number. A total of 3202 genomes were analyzed, and *M.tb* H37Rv was used as the reference genome sequence.

### Single nucleotide polymorphism (SNP) analysis

To map the sequencing reads to the standard isolate H37Rv, the BWA Mem algorithm (version 0.7.17-r1188) was used. We only included samples with a coverage rate of 98% or higher and a minimum depth of at least 20% [35]. Variant calling was performed using Samclip (version 0.4.0) and SAMtools (version 1.15), and the resulting variants were further filtered by Free Bayes (version 1.3.2) and Bcftools (version 1.15.1). We excluded Single nucleotide polymorphisms (SNPs) located in repeat regions, such as polymorphic GC-rich sequences (PE/PPE genes) and direct repeat SNPs, as well as repeat bases identified by Tandem Repeat Finder (version 4.09) and RepeatMask (version 4.1.2-P1) [36, 37]. Finally, the SNP was annotated with SnpEff v 4.1 l, and the result was obtained with Python programming language [38].

### Prediction of drug resistance

To identify drug resistance mutations, we compared known indels and SNPs using TBProfiler (version 2.8.12) and the tuberculosis database (TBDB) [39, 40]. We then searched for genotypic markers of drug resistance mutations in both first-line drugs (such as isoniazid, rifampicin, pyrazinamide, ethambutol, and streptomycin) and second-line drugs (such as ethionamide, quinolones, amikacin, capreomycin, and kanamycin), using a set of genetic polymorphisms. Mutations that were not correlated with phenotypic drug resistance were excluded as markers of genetic drug resistance [41]. For more information about the mutations detected as molecular resistance predictions in 3202 isolates, please refer to Supplementary Table 7.

### Phylogenetic analysis

The isolates were divided into different lineages according to Coll et al. [42] (Supplementary Tables 5–6). The maximum likelihood phylogenetic tree construction was performed using IQ-TREE (verdion1.6.12) using the JC nucleotide substitution model, the gamma model of rate heterogeneity, and 100 bootstrap replicates [43]. *M carneti* CIPT140010059 was considered an outlier, and five isolates belonging to two lineages were excluded. The phylogenetic tree was visualized by iTOL (https://itol.Embl.De/). However, isolates of lineage1were excluded from further analysis because of their small number. Therefore, a total of 3193 isolates were included in the final analysis.

### Propagation analysis

Cluster analysis was used to study the effect of fatty acid metabolism gene mutation on the transmission of *M.tb*. Clustering was defined as a group of isolates with less than 10 SNPs among each other (see Supplementary Table 8). To study the regional variations, the geographical location of the isolates in China was divided into seven natural regions. Then, the clusters were classified as cross-regional clusters or non-cross-regional clusters. The cross-regional cluster means that the strains in the cluster come from two or more different regions.

### Acquisition of fatty acid metabolic genes

According to the NCBI database, a total of 83 fatty acid metabolism genes were obtained. Mutations in genes involved in fatty acid metabolism were done by bcftools (version 1.15.1) with an included filter parameter ‘FMT/GT="1/1” && QUAL > = 100 && FMT/DP > = 10 && (FMT/AO)/(FMT/DP) > = 0’. The results were shown in Supplementary Table 9.

### Statistical analysis

The data are presented as a number (percent). The positions with mutation frequency < 0.01 in fatty acid metabolism genes were excluded from the analysis [44]. SPSS version 26 was used for statistical analysis. The comparison of categorical variables was done using the Pearson’s chi-square test or Fisher exact test as appropriate between clustered and non-clustered, as well as cross-regional and non-cross-regional clusters. Variables with univariate analysis were included in the binary logistic regression model for multivariate analysis. To analyze the effect of fatty acid metabolism gene mutations on cluster size, the rank correlation analysis of Spearman was carried out by using R version 4.1.0. All reported statistical tests were 2-sided, and *P* values < 0.05 were considered statistically significant.

### Electronic supplementary material

Below is the link to the electronic supplementary material.

**Supplementary Material 1: Supplement Table 1** Correlation analysis of fatty acid metabolism gene mutations between clustered and non-clustered isolates

**Supplementary Material 2: Supplement Table 2** Correlation analysis of fatty acid metabolism gene mutations between clustered and non-clustered isolates of lineage2

**Supplementary Material 3: Supplement Table 3** Correlation analysis of fatty acid metabolism gene mutations between clustered and non-clustered isolates of lineage4

**Supplementary Material 4: Supplement Table 4** Correlation analysis of fatty acid metabolism gene mutations between cross-regional and non-cross-regional clusters

**Supplementary Material 5: Supplement Table 5** Information about 1447 isolates of *Mycobacterium tuberculosis*

**Supplementary Material 6: Supplement Table 6** Information about 1755 isolates of *Mycobacterium tuberculosis*

**Supplementary Material 7: Supplement Table 7** Drug resistance of 3202 isolates of *Mycobacterium tuberculosis*

**Supplementary Material 8: Supplement Table 8** Information on clustering of 3193 isolates of *Mycobacterium tuberculosis*

**Supplementary Material 9: Supplement Table 9** Mutations of fatty acid metabolism genes in 3193 isolates of *Mycobacterium tuberculosis*

## Data Availability

The whole genome sequences have been submitted to the NCBI under the accession. number PRJNA1002108.

## Abbreviations

CTAB: Cetyltrimethylammonium Bromide
M.tb: Mycobacterium tuberculosis
QC: Quality control
SNP: Single nucleotide polymorphism
SNPs: Single nucleotide polymorphisms
SPHCC: Shandong Public Health Clinical Research Center
TB: Tuberculosis
TBDB: Tuberculosis database
WGS: Whole genome sequencing
WRCH: Weifang Respiratory Clinical Hospital
