# Correction: Association between fatty acid metabolism gene mutations and Mycobacterium tuberculosis transmission revealed by whole genome sequencing

**DOI:** 10.1186/s12866-023-03158-4

**Published:** 2024-01-03

**Authors:** Yameng Li, Xianglong Kong, Yifan Li, Ningning Tao, Tingting Wang, Yingying Li, Yawei Hou, Xuehan Zhu, Qilin Han, Yuzhen Zhang, Qiqi An, Yao Liu, Huaichen Li

**Affiliations:** 1 Shandong University of Traditional Chinese Medicine, Jinan, Shandong 250014 People’s Republic of China; 2 Artificial Intelligence Institute, Qilu University of Technology (Shandong Academy of Sciences), Jinan, Shandong 250011 People’s Republic of China; 3 Department of Respiratory and Critical Care Medicine, The Third Affiliated Hospital of Shandong First Medical University (Affiliated Hospital of Shandong Academy of Medical Sciences), Jinan, Shandong 250031 People’s Republic of China; 4 Department of Respiratory and Critical Care Medicine, Shandong Provincial Hospital, Shandong University, Shandong Provincial Hospital Affiliated to Shandong First Medical University, Jingwuweiqi Road, Huaiyin District, Jinan, Shandong 250021 People’s Republic of China; 5 Shandong First Medical University & Shandong Academy of Medical Sciences, Jinan, Shandong 250117 People’s Republic of China

**Correction:** ***BMC Microbiol*** **23, 379 (2023)**

10.1186/s12866-023-03072-9

Following publication of the original article [1], the author would like to correct the affiliation of the first author Yameng Li and the affiliation of the corresponding author Huaichen Li.

Therefore, the author has requested the publication of an erratum to address the following issues:

Incorrect: 1 Department of Chinese Medicine Integrated with Western Medicine, College of Traditional Chinese Medicine, Shandong University of Traditional Chinese Medicine, 16,369 Jingshi Road, Lixia District, Jinan, Shandong, 250,355, People’s Republic of China.

Corrected: 1 Shandong University of Traditional Chinese Medicine, Jinan, Shandong, 250,014, People’s Republic of China.
