# Supplementary material for: Association between fatty acid metabolism gene mutations and Mycobacterium tuberculosis transmission revealed by whole genome sequencing
Source: BMC Microbiol. 2023 Dec 1;23:379. doi: 10.1186/s12866-023-03072-9 (PMC10691062; doi:10.1186/s12866-023-03072-9)
Supplement: Supplementary file 1 — Supplementary Material 1: Supplement Table 1 Correlation analysis of fatty acid metabolism gene mutations between clustered and non-clustered isolates [file 12866_2023_3072_MOESM1_ESM.docx]

**Supplement Table1** Correlation analysis of fatty acid metabolism gene mutations between clustered and non-clustered isolates

| **Gene (position)** | **Category** | **Mutation** | **No mutation** | **Chi-square** | ***P* value** | **COR** |
| --- | --- | --- | --- | --- | --- | --- |
| ppiA (12555) | Cluster | 58(3.6) | 1538(96.4) | 26.622 | 2.474e-7 | -0.091 |
|  | Non-Cluster | 126(7.9) | 1471(92.1) |  |  |  |
| fadD34 (37305) | Cluster | 1457(91.3) | 139(8.7) | 82.457 | 1.080e-19 | 0.161 |
|  | Non-Cluster | 1278(80.0) | 319(20.0) |  |  |  |
| fadD34 (37334) | Cluster | 1142(71.6) | 454(28.4) | 33.316 | 7.888e-9 | 0.102 |
|  | Non-Cluster | 989(61.9) | 608(38.1) |  |  |  |
| fadD34 (37553) | Cluster | 39(2.4) | 1557(97.6) | 18.361 | 1.827e-5 | -0.076 |
|  | Non-Cluster | 86(5.4) | 1511(94.6) |  |  |  |
| fadD34 (37971) | Cluster | 23(1.4) | 1573(98.6) | 2.910 | 0.088 | -0.030 |
|  | Non-Cluster | 36(2.3) | 1561(97.7) |  |  |  |
| fadD34 (38199) | Cluster | 77(4.8) | 1519(95.2) | 0.000 | 0.997 | 0.000 |
|  | Non-Cluster | 77(4.8) | 1520(95.2) |  |  |  |
| gca (136605) | Cluster | 32(20.0) | 1564(98.0) | 29.445 | 5.753e-8 | 0.096 |
|  | Non-Cluster | 1(0.1) | 1596(99.9) |  |  |  |
| lipC (262829) | Cluster | 14(0.9) | 1582(99.1) | 4.167 | 0.041 | -0.036 |
|  | Non-Cluster | 27(1.7) | 1570(98.3) |  |  |  |
| clpB (460413) | Cluster | 1154(72.3) | 442(27.7) | 38.050 | 6.894e-10 | 0.109 |
|  | Non-Cluster | 991(62.1) | 606(37.9) |  |  |  |
| fgd1 (491556) | Cluster | 39(2.4) | 1557(97.6) | 0.710 | 0.399 | 0.015 |
|  | Non-Cluster | 32(20.0) | 1565(98.0) |  |  |  |
| fgd1 (491742) | Cluster | 1463(91.7) | 133(8.3) | 80.446 | 2.988e-19 | 0.159 |
|  | Non-Cluster | 1289(80.7) | 308(19.3) |  |  |  |
| proC (590436) | Cluster | 1596(100.0) | 0(0.0) | - | - | - |
|  | Non-Cluster | 1597(100.0) | 0(0.0) |  |  |  |
| pepC (893733) | Cluster | 1579(98.9) | 17(1.1) | 9.871 | 0.002 | -0.056 |
|  | Non-Cluster | 1594(99.8) | 3(0.2) |  |  |  |
| pepC (893895) | Cluster | 15(0.9) | 1581(99.1) | 7.470 | 0.006 | -0.048 |
|  | Non-Cluster | 34(2.1) | 1563(97.9) |  |  |  |
| far (951702) | Cluster | 1151(72.1) | 445(27.9) | 32.358 | 1.282e-8 | 0.101 |
|  | Non-Cluster | 1001(62.7) | 596(37.3) |  |  |  |
| fadB (957117) | Cluster | 1480(92.7) | 116(7.3) | 71.286 | 3.090e-17 | 0.149 |
|  | Non-Cluster | 1325(83.0) | 272(17.0) |  |  |  |
| ercc3 (958607) | Cluster | 23(1.4) | 1573(98.6) | 2.221 | 0.136 | 0.026 |
|  | Non-Cluster | 14(0.9) | 1583(99.1) |  |  |  |
| ercc3 (959167) | Cluster | 15(0.9) | 1581(99.1) | 0.274 | 0.601 | -0.009 |
|  | Non-Cluster | 18(1.1) | 1579(98.9) |  |  |  |
| pepD (1100234) | Cluster | 1596(100.0) | 0(0.0) | - | - | - |
|  | Non-Cluster | 1597(100.0) | 0(0.0) |  |  |  |
| arcA (1117201) | Cluster | 15(0.9) | 1581(99.1) | 7.470 | 0.006 | -0.048 |
|  | Non-Cluster | 34(2.1) | 1563(97.9) |  |  |  |

**Supplement Table1**(Continue)

| **Gene (position)** | **Category** | **Mutation** | **No mutation** | **Chi-square** | ***P* value** | **COR** |
| --- | --- | --- | --- | --- | --- | --- |
| fadH (1306259) | Cluster | 1478(92.6) | 118(7.4) | 69.909 | 6.212e-17 | 0.148 |
|  | Non-Cluster | 1324(82.9) | 273(17.1) |  |  |  |
| fadH (1306322) | Cluster | 21(1.3) | 1575(98.7) | 2.705 | 0.100 | -0.029 |
|  | Non-Cluster | 33(2.1) | 1564(97.9) |  |  |  |
| fadH (1306796) | Cluster | 15(0.9) | 1581(99.1) | 8.774 | 0.003 | -0.052 |
|  | Non-Cluster | 36(2.3) | 1561(97.7) |  |  |  |
| fadH (1307598) | Cluster | 1594(99.9) | 2(0.1) | 1.287 | 0.257 | 0.020 |
|  | Non-Cluster | 1592(99.7) | 5(0.3) |  |  |  |
| ogt (1477346) | Cluster | 25(1.6) | 1571(98.4) | 6.509 | 0.011 | 0.045 |
|  | Non-Cluster | 10(0.6) | 1587(99.4) |  |  |  |
| ogt (1477522) | Cluster | 130(8.1) | 1466(91.9) | 0.110 | 0.740 | 0.006 |
|  | Non-Cluster | 125(7.8) | 1472(92.2) |  |  |  |
| ogt (1477596) | Cluster | 1154(72.3) | 442(27.7) | 38.498 | 5.482e-10 | 0.110 |
|  | Non-Cluster | 990(62.0) | 607(38.0) |  |  |  |
| lipI (1576481) | Cluster | 1397(87.5) | 199(12.5) | 92.408 | 7.053e-22 | 0.170 |
|  | Non-Cluster | 1184(74.1) | 413(25.9) |  |  |  |
| lipI (1576527) | Cluster | 1400(87.7) | 196(12.3) | 90.859 | 1.543e-21 | 0.169 |
|  | Non-Cluster | 1190(74.5) | 407(25.5) |  |  |  |
| tkt (1630148) | Cluster | 1596(100.0) | 0(0.0) | 3.001 | 0.083 | 0.031 |
|  | Non-Cluster | 1594(99.8) | 3(0.2) |  |  |  |
| inhA (1674210) | Cluster | 50(3.1) | 1546(96.9) | 1.122 | 0.289 | -0.019 |
|  | Non-Cluster | 61(3.8) | 1536(96.2) |  |  |  |
| fadD11 (1754459) | Cluster | 14(0.9) | 1582(99.1) | 3.638 | 0.056 | -0.034 |
|  | Non-Cluster | 26(1.6) | 1571(98.4) |  |  |  |
| lgt (1814428) | Cluster | 23(1.4) | 1573(98.6) | 4.201 | 0.040 | -0.036 |
|  | Non-Cluster | 39(2.4) | 1558(97.6) |  |  |  |
| rpsA (1834177) | Cluster | 1461(91.5) | 135(8.5) | 84.457 | 3.927e-20 | 0.163 |
|  | Non-Cluster | 1281(80.2) | 316(19.8) |  |  |  |
| rpsA (1834776) | Cluster | 29(1.8) | 1567(98.2) | 17.139 | 3.475e-5 | 0.073 |
|  | Non-Cluster | 5(0.3) | 1592(99.7) |  |  |  |
| tlyA (1917972) | Cluster | 1594(99.9) | 2(0.1) | 2.003 | 0.157 | -0.025 |
|  | Non-Cluster | 1597(100.0) | 0(0.0) |  |  |  |
| lipJ (2146429) | Cluster | 15(0.9) | 1581(99.1) | 7.470 | 0.006 | -0.048 |
|  | Non-Cluster | 34(2.1) | 1563(97.9) |  |  |  |
| lipJ (2147022) | Cluster | 1592(99.7) | 4(0.3) | 0.819 | 0.365 | 0.016 |
|  | Non-Cluster | 1590(99.6) | 7(0.4) |  |  |  |
| helZ (2361030) | Cluster | 14(0.9) | 1582(99.1) | 0.503 | 0.478 | -0.013 |
|  | Non-Cluster | 18(1.1) | 1579(98.9) |  |  |  |
| helZ (2361257) | Cluster | 32(20.0) | 1564(98.0) | 29.445 | 5.753e-8 | 0.096 |
|  | Non-Cluster | 1(0.1) | 1596(99.9) |  |  |  |

**Supplement Table1**(Continue)

| **Gene (position)** | **Category** | **Mutation** | **No mutation** | **Chi-square** | ***P* value** | **COR** |
| --- | --- | --- | --- | --- | --- | --- |
| helZ (2361311) | Cluster | 57(3.6) | 1539(96.4) | 26.897 | 2.146e-7 | -0.092 |
|  | Non-Cluster | 125(7.8) | 1472(92.2) |  |  |  |
| helZ (2361492) | Cluster | 15(0.9) | 1581(99.1) | 7.470 | 0.006 | -0.048 |
|  | Non-Cluster | 34(2.1) | 1563(97.9) |  |  |  |
| helZ (2361604) | Cluster | 1590(99.6) | 6(0.4) | 0.286 | 0.593 | 0.009 |
|  | Non-Cluster | 1589(99.5) | 8(0.5) |  |  |  |
| helZ (2362041) | Cluster | 1596(100.0) | 0(0.0) | 3.203 | 0.074 | 0.040 |
|  | Non-Cluster | 1592(99.7) | 5(0.3) |  |  |  |
| fadD15 (2448458) | Cluster | 1465(91.8) | 131(8.2) | 82.612 | 9.986e-20 | 0.161 |
|  | Non-Cluster | 1289(80.7) | 308(19.3) |  |  |  |
| fadD15 (2449629) | Cluster | 46(2.9) | 1550(97.1) | 20.321 | 6.546e-6 | 0.080 |
|  | Non-Cluster | 12(0.8) | 1585(99.2) |  |  |  |
| dlaT (2482888) | Cluster | 15(0.9) | 1581(99.1) | 6.842 | 0.009 | -0.046 |
|  | Non-Cluster | 33(2.1) | 1564(97.9) |  |  |  |
| ptpA (2507412) | Cluster | 15(0.9) | 1581(99.1) | 7.470 | 0.006 | -0.048 |
|  | Non-Cluster | 34(2.1) | 1563(97.9) |  |  |  |
| acpS (2839689) | Cluster | 82(5.1) | 1514(94.9) | 0.098 | 0.754 | -0.006 |
|  | Non-Cluster | 86(5.4) | 1511(94.6) |  |  |  |
| fas (2841022) | Cluster | 1458(91.4) | 138(8.6) | 75.940 | 2.924e-18 | 0.154 |
|  | Non-Cluster | 1288(80.7) | 309(19.3) |  |  |  |
| fas (2847281) | Cluster | 1464(91.7) | 132(8.3) | 83.071 | 7.915e-20 | 0.161 |
|  | Non-Cluster | 1287(80.6) | 310(19.4) |  |  |  |
| relA (2908252) | Cluster | 61(3.8) | 1535(96.2) | 13.531 | 2.347e-4 | 0.065 |
|  | Non-Cluster | 27(1.7) | 1570(98.3) |  |  |  |
| arsA (3001498) | Cluster | 24(1.5) | 1572(98.5) | 4.528 | 0.033 | -0.038 |
|  | Non-Cluster | 41(2.6) | 1556(97.4) |  |  |  |
| arsA (3001785) | Cluster | 14(0.9) | 1582(99.1) | 1.066 | 0.302 | -0.018 |
|  | Non-Cluster | 20(1.3) | 1577(98.7) |  |  |  |
| ugpA (3141585) | Cluster | 14(0.9) | 1582(99.1) | 3.638 | 0.056 | -0.034 |
|  | Non-Cluster | 26(1.6) | 1571(98.4) |  |  |  |
| mtr (3165636) | Cluster | 1188(74.4) | 408(25.6) | 42.251 | 8.027e-11 | 0.115 |
|  | Non-Cluster | 1019(63.8) | 578(36.2) |  |  |  |
| tesA (3242617) | Cluster | 32(20.0) | 1564(98) | 0.636 | 0.425 | 0.014 |
|  | Non-Cluster | 26(1.6) | 1571(98.4) |  |  |  |
| gatB (3367765) | Cluster | 1595(99.9) | 1(0.1) | 1.001 | 0.317 | -0.018 |
|  | Non-Cluster | 1597(100.0) | 0(0.0) |  |  |  |
| cstA (3428183) | Cluster | 75(4.7) | 1521(95.3) | 0.066 | 0.797 | 0.005 |
|  | Non-Cluster | 72(4.5) | 1525(95.5) |  |  |  |
| cstA (3428917) | Cluster | 1592(99.7) | 4(0.3) | 2.255 | 0.133 | -0.035 |
|  | Non-Cluster | 1597(100.0) | 0(0.0) |  |  |  |

**Supplement Table1**(Continue)

| **Gene (position)** | **Category** | **Mutation** | **No mutation** | **Chi-square** | ***P* value** | **COR** |
| --- | --- | --- | --- | --- | --- | --- |
| agpS (3476350) | Cluster | 53(3.3) | 1543(96.7) | 0.515 | 0.473 | 0.013 |
|  | Non-Cluster | 46(2.9) | 1551(97.1) |  |  |  |
| nudC (3571828) | Cluster | 1397(87.5) | 199(12.5) | 89.430 | 3.176e-21 | 0.167 |
|  | Non-Cluster | 1188(74.4) | 409(25.6) |  |  |  |
| sdhD (3704596) | Cluster | 1596(100.0) | 0(0.0) | - | - | - |
|  | Non-Cluster | 1597(100.0) | 0(0.0) |  |  |  |
| sdhD (3704686) | Cluster | 58(3.6) | 1538(96.4) | 35.440 | 2.630e-9 | -0.105 |
|  | Non-Cluster | 139(8.7) | 1458(91.3) |  |  |  |
| sdhD (3704770) | Cluster | 1461(91.5) | 135(8.5) | 99.649 | 1.819e-23 | 0.177 |
|  | Non-Cluster | 1262(79.0) | 335(21.0) |  |  |  |
| nagA (3719723) | Cluster | 17(1.1) | 1579(98.9) | 12.878 | 3.325e-4 | -0.064 |
|  | Non-Cluster | 45(2.8) | 1552(97.2) |  |  |  |
| lipF (3906311) | Cluster | 1459(91.4) | 137(8.6) | 98.952 | 2.586e-23 | 0.176 |
|  | Non-Cluster | 1260(78.9) | 337(21.1) |  |  |  |
| lipF (3906881) | Cluster | 15(0.9) | 1581(99.1) | 0.274 | 0.601 | -0.009 |
|  | Non-Cluster | 18(1.1) | 1579(98.9) |  |  |  |
| acs (4108495) | Cluster | 33(2.1) | 1563(97.9) | 1.824 | 0.177 | 0.024 |
|  | Non-Cluster | 23(1.4) | 1574(98.6) |  |  |  |
| acs (4109342) | Cluster | 70(4.4) | 1526(95.6) | 0.651 | 0.420 | 0.014 |
|  | Non-Cluster | 61(3.8) | 1536(96.2) |  |  |  |
| crp (4116610) | Cluster | 1324(83.0) | 272(17.0) | 58.315 | 2.233e-14 | 0.135 |
|  | Non-Cluster | 1144(71.6) | 453(28.4) |  |  |  |
| crp (4116773) | Cluster | 49(3.1) | 1547(96.9) | 45.721 | 1.364e-11 | -0.120 |
|  | Non-Cluster | 139(8.7) | 1458(91.3) |  |  |  |
| pcnA (4392373) | Cluster | 21(1.3) | 1575(98.7) | 16.280 | 5.464e-5 | -0.071 |
|  | Non-Cluster | 56(3.5) | 1541(96.5) |  |  |  |

COR, correlation coefficient.

-means there is no result in statistical software or the result was too large and nonsense.
