# Supplementary material for: Association between fatty acid metabolism gene mutations and Mycobacterium tuberculosis transmission revealed by whole genome sequencing
Source: BMC Microbiol. 2023 Dec 1;23:379. doi: 10.1186/s12866-023-03072-9 (PMC10691062; doi:10.1186/s12866-023-03072-9)
Supplement: Supplementary file 2 — Supplementary Material 2: Supplement Table 2 Correlation analysis of fatty acid metabolism gene mutations between clustered and non-clustered isolates of lineage2 [file 12866_2023_3072_MOESM2_ESM.docx]

**Supplement Table2** Correlation analysis of fatty acid metabolism gene mutations between clustered and non-clustered isolates of lineage2

| **Gene (position)** | **Category** | **Mutation** | **No mutation** | **Chi-square** | ***P* value** | **COR** |
| --- | --- | --- | --- | --- | --- | --- |
| fadD34 (37305) | Cluster | 1457(99.59) | 6(0.41) | 0.647 | 0.421 | -0.015 |
|  | Non-Cluster | 1278(99.77) | 3(0.23) |  |  |  |
| fadD34 (37334) | Cluster | 1142(78.06) | 321(21.94) | 0.287 | 0.592 | 0.010 |
|  | Non-Cluster | 989(77.21) | 292(22.79) |  |  |  |
| fadD34 (37971) | Cluster | 23(1.57) | 1440(98.43) | 4.977 | 0.026 | -0.043 |
|  | Non-Cluster | 36(2.81) | 1245(97.19) |  |  |  |
| fadD34 (38199) | Cluster | 77(5.26) | 1386(94.74) | 0.721 | 0.396 | -0.016 |
|  | Non-Cluster | 77(6.01) | 1204(93.99) |  |  |  |
| gca (136605) | Cluster | 32(2.19) | 1431(97.81) | 25.573 | 4.260e-7 | 0.097 |
|  | Non-Cluster | 1(0.08) | 1280(99.92) |  |  |  |
| lipC (262829) | Cluster | 14(0.96) | 1449(99.04) | 6.145 | 0.013 | -0.047 |
|  | Non-Cluster | 27(2.11) | 1254(97.89) |  |  |  |
| clpB (460413) | Cluster | 1154(78.88) | 309(21.12) | 0.922 | 0.337 | 0.018 |
|  | Non-Cluster | 991(77.36) | 290(22.64) |  |  |  |
| fgd1 (491556) | Cluster | 39(2.67) | 1424(97.33) | 0.076 | 0.782 | 0.005 |
|  | Non-Cluster | 32(2.5) | 1249(97.5) |  |  |  |
| fgd1 (491742) | Cluster | 1461(99.86) | 2(0.14) | - | 0.502 | -0.025 |
|  | Non-Cluster | 1281(10) | 0(0.0) |  |  |  |
| proC (590436) | Cluster | 1463(10) | 0(0.0) | - | - | - |
|  | Non-Cluster | 1281(10) | 0(0.0) |  |  |  |
| pepC (893733) | Cluster | 1447(98.91) | 16(1.09) | 7.337 | 0.007 | -0.052 |
|  | Non-Cluster | 1278(99.77) | 3(0.23) |  |  |  |
| far (951702) | Cluster | 1151(78.67) | 312(21.33) | 0.114 | 0.735 | 0.006 |
|  | Non-Cluster | 1001(78.14) | 280(21.86) |  |  |  |
| fadB (957117) | Cluster | 1463(10) | 0(0.0) | - | - | - |
|  | Non-Cluster | 1281(10) | 0(0.0) |  |  |  |
| ercc3 (958607) | Cluster | 23(1.57) | 1440(98.43) | 1.179 | 0.278 | 0.021 |
|  | Non-Cluster | 14(1.09) | 1267(98.91) |  |  |  |
| pepD (1100234) | Cluster | 1463(10) | 0(0.0) | - | - | 0.000 |
|  | Non-Cluster | 1281(10) | 0(0.0) |  |  |  |
| fadH (1306259) | Cluster | 1461(99.86) | 2(0.14) | 0.000 | 1.000 | -0.009 |
|  | Non-Cluster | 1280(99.92) | 1(0.08) |  |  |  |
| fadH (1307598) | Cluster | 1462(99.93) | 1(0.07) | - | 1.000 | 0.002 |
|  | Non-Cluster | 1280(99.92) | 1(0.08) |  |  |  |
| ogt (1477346) | Cluster | 25(1.71) | 1438(98.29) | 4.673 | 0.031 | 0.041 |
|  | Non-Cluster | 10(0.78) | 1271(99.22) |  |  |  |
| ogt (1477522) | Cluster | 130(8.89) | 1333(91.11) | 0.616 | 0.432 | -0.015 |
|  | Non-Cluster | 125(9.76) | 1156(90.24) |  |  |  |
| ogt (1477596) | Cluster | 1154(78.88) | 309(21.12) | 1.018 | 0.313 | 0.019 |
|  | Non-Cluster | 990(77.28) | 291(22.72) |  |  |  |

**Supplement Table2**(Continue)

| **Gene (position)** | **Category** | **Mutation** | **No mutation** | **Chi-square** | ***P* value** | **COR** |
| --- | --- | --- | --- | --- | --- | --- |
| lipI (1576481) | Cluster | 1397(95.49) | 66(4.51) | 11.453 | 0.001 | 0.065 |
|  | Non-Cluster | 1184(92.43) | 97(7.57) |  |  |  |
| lipI (1576527) | Cluster | 1400(95.69) | 63(4.31) | 10.091 | 0.001 | 0.061 |
|  | Non-Cluster | 1190(92.9) | 91(7.1) |  |  |  |
| tkt (1630148) | Cluster | 1463(10) | 0(0.0) | - | - | - |
|  | Non-Cluster | 1281(10) | 0(0.0) |  |  |  |
| inhA (1674210) | Cluster | 50(3.42) | 1413(96.58) | 3.180 | 0.075 | -0.034 |
|  | Non-Cluster | 61(4.76) | 1220(95.24) |  |  |  |
| fadD11 (1754459) | Cluster | 14(0.96) | 1449(99.04) | 5.471 | 0.019 | -0.045 |
|  | Non-Cluster | 26(2.03) | 1255(97.97) |  |  |  |
| lgt (1814428) | Cluster | 23(1.57) | 1440(98.43) | 6.705 | 0.010 | -0.049 |
|  | Non-Cluster | 39(3.04) | 1242(96.96) |  |  |  |
| rpsA (1834177) | Cluster | 1461(99.86) | 2(0.14) | - | 0.502 | -0.025 |
|  | Non-Cluster | 1281(10) | 0(0.0) |  |  |  |
| rpsA (1834776) | Cluster | 29(1.98) | 1434(98.02) | 14.144 | 1.694e-4 | 0.072 |
|  | Non-Cluster | 5(0.39) | 1276(99.61) |  |  |  |
| tlyA (1917972) | Cluster | 1461(99.86) | 2(0.14) | - | 0.502 | -0.025 |
|  | Non-Cluster | 1281(10) | 0(0.0) |  |  |  |
| lipJ (2147022) | Cluster | 1459(99.73) | 4(0.27) | 0.000 | 1.000 | -0.004 |
|  | Non-Cluster | 1278(99.77) | 3(0.23) |  |  |  |
| helZ (2361257) | Cluster | 32(2.19) | 1431(97.81) | 25.573 | 4.260e-7 | 0.097 |
|  | Non-Cluster | 1(0.08) | 1280(99.92) |  |  |  |
| helZ (2361604) | Cluster | 1458(99.66) | 5(0.34) | 0.028 | 0.868 | -0.010 |
|  | Non-Cluster | 1278(99.77) | 3(0.23) |  |  |  |
| helZ (2362041) | Cluster | 1463(10) | 0(0.0) | 1.621 | 0.203 | 0.035 |
|  | Non-Cluster | 1278(99.77) | 3(0.23) |  |  |  |
| fadD15 (2448458) | Cluster | 1463(10) | 0(0.0) | - | - | - |
|  | Non-Cluster | 1281(10) | 0(0.0) |  |  |  |
| fadD15 (2449629) | Cluster | 46(3.14) | 1417(96.86) | 16.085 | 6.055e-5 | 0.077 |
|  | Non-Cluster | 12(0.94) | 1269(99.06) |  |  |  |
| acpS (2839689) | Cluster | 82(5.6) | 1381(94.4) | 1.269 | 0.260 | -0.022 |
|  | Non-Cluster | 85(6.64) | 1196(93.36) |  |  |  |
| fas (2841022) | Cluster | 1456(99.52) | 7(0.48) | 2.515 | 0.113 | -0.037 |
|  | Non-Cluster | 1280(99.92) | 1(0.08) |  |  |  |
| fas (2847281) | Cluster | 1462(99.93) | 1(0.07) | 0.013 | 0.908 | 0.013 |
|  | Non-Cluster | 1279(99.84) | 2(0.16) |  |  |  |
| relA (2908252) | Cluster | 61(4.17) | 1402(95.83) | 9.353 | 0.002 | 0.058 |
|  | Non-Cluster | 27(2.11) | 1254(97.89) |  |  |  |
| arsA (3001498) | Cluster | 24(1.64) | 1439(98.36) | 7.188 | 0.007 | -0.051 |
|  | Non-Cluster | 41(3.2) | 1240(96.8) |  |  |  |

**Supplement Table2**(Continue)

| **Gene (position)** | **Category** | **Mutation** | **No mutation** | **Chi-square** | ***P* value** | **COR** |
| --- | --- | --- | --- | --- | --- | --- |
| arsA (3001785) | Cluster | 14(0.96) | 1449(99.04) | 2.038 | 0.153 | -0.027 |
|  | Non-Cluster | 20(1.56) | 1261(98.44) |  |  |  |
| ugpA (3141585) | Cluster | 14(0.96) | 1449(99.04) | 5.471 | 0.019 | -0.045 |
|  | Non-Cluster | 26(2.03) | 1255(97.97) |  |  |  |
| mtr (3165636) | Cluster | 1188(81.2) | 275(18.8) | 1.190 | 0.275 | 0.021 |
|  | Non-Cluster | 1019(79.55) | 262(20.45) |  |  |  |
| tesA (3242617) | Cluster | 32(2.19) | 1431(97.81) | 0.082 | 0.775 | 0.005 |
|  | Non-Cluster | 26(2.03) | 1255(97.97) |  |  |  |
| gatB (3367765) | Cluster | 1463(10) | 0(0.0) | - | - | - |
|  | Non-Cluster | 1281(10) | 0(0.0) |  |  |  |
| cstA (3428183) | Cluster | 147(5.36) | 2597(94.64) | 0.329 | 0.566 | -0.011 |
|  | Non-Cluster | 75(5.13) | 1388(94.87) |  |  |  |
| cstA (3428917) | Cluster | 2742(99.93) | 2(0.07) | - | 0.502 | -0.025 |
|  | Non-Cluster | 1461(99.86) | 2(0.14) |  |  |  |
| agpS (3476350) | Cluster | 53(3.62) | 1410(96.38) | 0.002 | 0.965 | 0.001 |
|  | Non-Cluster | 46(3.59) | 1235(96.41) |  |  |  |
| nudC (3571828) | Cluster | 1397(95.5) | 66(4.5) | 9.453 | 0.002 | 0.059 |
|  | Non-Cluster | 1188(92.7) | 93(7.3) |  |  |  |
| sdhD (3704596) | Cluster | 1463(10) | 0(0.0) | - | - | 0.000 |
|  | Non-Cluster | 1281(10) | 0(0.0) |  |  |  |
| lipF (3906311) | Cluster | 1459(99.73) | 4(0.27) | 14.115 | 1.719e-4 | 0.072 |
|  | Non-Cluster | 1260(98.36) | 21(1.64) |  |  |  |
| acs (4108495) | Cluster | 33(2.26) | 1430(97.74) | 0.723 | 0.395 | 0.016 |
|  | Non-Cluster | 23(1.8) | 1258(98.2) |  |  |  |
| acs (4109342) | Cluster | 70(4.78) | 1393(95.22) | 0.001 | 0.978 | 0.001 |
|  | Non-Cluster | 61(4.76) | 1220(95.24) |  |  |  |
| crp (4116610) | Cluster | 1324(90.5) | 139(9.5) | 1.076 | 0.300 | 0.020 |
|  | Non-Cluster | 1144(89.31) | 137(10.69) |  |  |  |
| sdhD (3704770) | Cluster | 1461(99.9) | 2(0.1) | 16.305 | 5.391e-5 | 0.077 |
|  | Non-Cluster | 1262(98.5) | 19(1.5) |  |  |  |

COR, correlation coefficient.

-means there is no result in statistical software or the result was too large and nonsense.
