# Supplementary material for: Association between fatty acid metabolism gene mutations and Mycobacterium tuberculosis transmission revealed by whole genome sequencing
Source: BMC Microbiol. 2023 Dec 1;23:379. doi: 10.1186/s12866-023-03072-9 (PMC10691062; doi:10.1186/s12866-023-03072-9)
Supplement: Supplementary file 3 — Supplementary Material 3: Supplement Table 3 Correlation analysis of fatty acid metabolism gene mutations between clustered and non-clustered isolates of lineage4 [file 12866_2023_3072_MOESM3_ESM.docx]

**Supplement Table3** Correlation analysis of fatty acid metabolism gene mutations between clustered and non-clustered isolates of lineage4

| **Gene (position)** | **Category** | **Mutation** | **No mutation** | **Chi-square** | ***P* value** | **COR** |
| --- | --- | --- | --- | --- | --- | --- |
| ppiA（12555) | Cluster | 58(44.30) | 73(55.7) | 0.428 | 0.513 | 0.031 |
|  | Non-cluster | 126(40.9) | 182(59.1) |  |  |  |
| fadD34（37553) | Cluster | 39(29.8) | 92(70.2) | 0.154 | 0.694 | 0.019 |
|  | Non-cluster | 86(27.9) | 222(72.1) |  |  |  |
| proC (590436) | Cluster | 131(10) | 0(0.0) | - | - | - |
|  | Non-cluster | 308(10) | 0(0.0) |  |  |  |
| pepC（893733) | Cluster | 130(99.2) | 1(0.8) | - | 0.298 | -0.073 |
|  | Non-cluster | 308(100.0) | 0(0.0) |  |  |  |
| pepC（893895) | Cluster | 15(11.5) | 116(88.5) | 0.016 | 0.900 | 0.006 |
|  | Non-cluster | 34(11.0) | 274(89.0) |  |  |  |
| fadB（957117) | Cluster | 15(11.5) | 116(88.5) | 0.005 | 0.943 | -0.003 |
|  | Non-cluster | 36(11.7) | 272(88.3) |  |  |  |
| ercc3（959167) | Cluster | 15(11.5) | 116(88.5) | 4.115 | 0.042 | 0.097 |
|  | Non-cluster | 18(5.8) | 290(94.2) |  |  |  |
| pepD（1100234) | Cluster | 131(100.0) | 0(0.0) | - | - | -. |
|  | Non-cluster | 308(100.0) | 0(0.0) |  |  |  |
| arcA（1117201) | Cluster | 15(11.5) | 116(88.5) | 0.016 | 0.900 | 0.006 |
|  | Non-cluster | 34(11.0) | 274(89.0) |  |  |  |
| fadH（1306259) | Cluster | 15(11.5) | 116(88.5) | 0.005 | 0.943 | -0.003 |
|  | Non-cluster | 36(11.7) | 272(88.3) |  |  |  |
| fadH（1306322) | Cluster | 21(16.0) | 110(84.0) | 2.408 | 0.121 | 0.074 |
|  | Non-cluster | 33(10.7) | 275(89.3) |  |  |  |
| fadH（1306796) | Cluster | 15(11.5) | 116(88.5) | 0.005 | 0.943 | -0.003 |
|  | Non-cluster | 36(11.7) | 272(88.3) |  |  |  |
| fadH（1307598) | Cluster | 130(99.2) | 1(0.8) | 0.000 | 1.000 | 0.023 |
|  | Non-cluster | 304(98.7) | 4(1.3) |  |  |  |
| tkt（1630148) | Cluster | 131(100.0) | 0(0.0) | - | 0.558 | 0.054 |
|  | Non-cluster | 305(99.0) | 3(1.0) |  |  |  |
| tlyA（1917972) | Cluster | 131(100.0) | 0(0.0) | - | - | - |
|  | Non-cluster | 308(100.0) | 0(0.0) |  |  |  |
| lipJ（2146429) | Cluster | 15(11.5) | 116(88.5) | 0.016 | 0.900 | 0.006 |
|  | Non-cluster | 34(11.0) | 274(89.0) |  |  |  |
| lipJ（2147022) | Cluster | 131(100.0) | 0(0.0) | 0.580 | 0.446 | 0.063 |
|  | Non-cluster | 304(98.7) | 4(1.3) |  |  |  |
| helZ（2361030) | Cluster | 14(9.7) | 117(89.3) | 3.190 | 0.074 | 0.085 |
|  | Non-cluster | 18(5.8) | 290(94.2) |  |  |  |
| helZ（2361311) | Cluster | 57(43.5) | 74(56.5) | 0.324 | 0.569 | 0.027 |
|  | Non-cluster | 125(40.6) | 183(59.4) |  |  |  |
| helZ（2361492) | Cluster | 15(11.5) | 116(88.5) | 0.016 | 0.900 | 0.006 |
|  | Non-cluster | 34(11.0) | 274(89.0) |  |  |  |

**Supplement Table3**(Continue)

| **Gene (position)** | **Category** | **Mutation** | **No mutation** | **Chi-square** | ***P* value** | **COR** |
| --- | --- | --- | --- | --- | --- | --- |
| helZ（2361604) | Cluster | 130(99.2) | 1(0.8) | 0.068 | 0.794 | 0.034 |
|  | Non-cluster | 303(98.4) | 5(1.6) |  |  |  |
| helZ（2362041) | Cluster | 131(100.0) | 0(0.0) | - | 1.000 | 0.044 |
|  | Non-cluster | 306(99.4) | 2(0.6) |  |  |  |
| dlaT（2482888) | Cluster | 15(11.5) | 116(88.5) | 0.051 | 0.821 | 0.011 |
|  | Non-cluster | 33(10.7) | 275(89.3) |  |  |  |
| ptpA（2507412) | Cluster | 15(11.5) | 116(88.5) | 0.016 | 0.900 | 0.006 |
|  | Non-cluster | 34(11.0) | 274(89.0) |  |  |  |
| acpS（2839689) | Cluster | 0(0.0) | 131(100.0) | - | 1.000 | -0.031 |
|  | Non-cluster | 1(0.3) | 307(99.7) |  |  |  |
| gatB（3367765) | Cluster | 131(99.2) | 1(0.8) | - | 0.298 | -0.073 |
|  | Non-cluster | 308(100.0) | 0(0.0) |  |  |  |
| cstA（3428917) | Cluster | 129(98.5) | 2(1.5) | - | 0.089 | -0.104 |
|  | Non-cluster | 308(100.0) | 0(0.0) |  |  |  |
| sdhD（3704596) | Cluster | 131(100.0) | 0(0.0) | - | - | -. |
|  | Non-cluster | 308(100.0) | 0(0.0) |  |  |  |
| sdhD（3704686) | Cluster | 58(44.3) | 73(55.7) | 0.027 | 0.869 | -0.008 |
|  | Non-cluster | 139(45.1) | 169(54.9) |  |  |  |
| nagA（3719723) | Cluster | 17(13.0) | 114(87.0) | 0.202 | 0.653 | -0.021 |
|  | Non-cluster | 45(14.6) | 263(85.4) |  |  |  |
| lipF（3906881) | Cluster | 15(11.5) | 116(88.5) | 4.155 | 0.042 | 0.097 |
|  | Non-cluster | 18(5.8) | 290(94.2) |  |  |  |
| crp（4116773) | Cluster | 49(37.4) | 82(62.6) | 2.240 | 0.134 | -0.071 |
|  | Non-cluster | 139(45.1) | 169(54.9) |  |  |  |
| pcnA(4392373) | Cluster | 21(16.0) | 110(84.0) | 0.294 | 0.585 | -0.026 |
|  | Non-cluster | 56(18.2) | 252(81.8) |  |  |  |

COR, correlation coefficient.

-means there is no result in statistical software or the result was too large and nonsense.
