# Supplementary material for: Association between fatty acid metabolism gene mutations and Mycobacterium tuberculosis transmission revealed by whole genome sequencing
Source: BMC Microbiol. 2023 Dec 1;23:379. doi: 10.1186/s12866-023-03072-9 (PMC10691062; doi:10.1186/s12866-023-03072-9)
Supplement: Supplementary file 4 — Supplementary Material 4: Supplement Table 4 Correlation analysis of fatty acid metabolism gene mutations between cross-regional and non-cross-regional clusters [file 12866_2023_3072_MOESM4_ESM.docx]

**Supplement Table4** Correlation analysis of fatty acid metabolism gene mutations between cross-regional and non-cross-regional clusters

| **Gene (position)** | **Category** | **Mutation** | **No mutation** | **Chi-square** | ***P* value** | **COR** |
| --- | --- | --- | --- | --- | --- | --- |
| ppiA (12555) | cross-regional | 8(2.0) | 399(98.0) | 4.343 | 0.037 | -0.052 |
|  | Non-cross-regional | 50(4.2) | 1139(95.8) |  |  |  |
| fadD34 (37305) | cross-regional | 383(94.1) | 24(5.9) | 5.435 | 0.020 | 0.058 |
|  | Non-cross-regional | 1074(90.3) | 115(9.7) |  |  |  |
| fadD34 (37334) | cross-regional | 274(67.3) | 133(32.7) | 4.807 | 0.028 | -0.055 |
|  | Non-cross-regional | 868(73.0) | 321(27.0) |  |  |  |
| fadD34 (37553) | cross-regional | 9(2.2) | 398(97.8) | 0.124 | 0.725 | -0.009 |
|  | Non-cross-regional | 30(2.5) | 1159(97.5) |  |  |  |
| fadD34 (37971) | cross-regional | 8(2.0) | 399(98.0) | 1.058 | 0.304 | 0.026 |
|  | Non-cross-regional | 15(1.3) | 1174(98.7) |  |  |  |
| fadD34 (38199) | cross-regional | 21(5.2) | 386(94.8) | 0.134 | 0.715 | 0.009 |
|  | Non-cross-regional | 56(4.7) | 1133(95.3) |  |  |  |
| gca (136605) | cross-regional | 0(0.0) | 407(100.0) | 11.178 | 0.001 | -0.084 |
|  | Non-cross-regional | 32(2.7) | 1157(97.3) |  |  |  |
| lipC (262829) | cross-regional | 10(2.5) | 397(97.5) | 13.337 | 7.496e-5 | 0.099 |
|  | Non-cross-regional | 4(0.3) | 1185(99.7) |  |  |  |
| clpB (460413) | cross-regional | 274(67.3) | 133(32.7) | 6.777 | 0.009 | -0.065 |
|  | Non-cross-regional | 880(74.0) | 309(26.0) |  |  |  |
| fgd1 (491556) | cross-regional | 10(2.5) | 397(97.5) | 0.000 | 0.984 | 0.001 |
|  | Non-cross-regional | 29(2.4) | 1160(97.6) |  |  |  |
| fgd1 (491742) | cross-regional | 383(94.1) | 24(5.9) | 4.246 | 0.039 | 0.052 |
|  | Non-cross-regional | 1080(90.8) | 109(9.2) |  |  |  |
| proC (590436) | cross-regional | 407(100.0) | 0(0.0) | - | - | -. |
|  | Non-cross-regional | 1189(100.0) | 0(0.0) |  |  |  |
| pepC (893733) | cross-regional | 399(98.0) | 8(2.0) | 3.135 | 0.177 | -0.051 |
|  | Non-cross-regional | 1180(99.2) | 9(0.8) |  |  |  |
| far (951702) | cross-regional | 278(68.3) | 129(31.7) | 3.950 | 0.047 | -0.050 |
|  | Non-cross-regional | 873(73.4) | 316(26.6) |  |  |  |
| fadB (957117) | cross-regional | 387(95.1) | 20(4.9) | 4.492 | 0.034 | 0.053 |
|  | Non-cross-regional | 1093(91.9) | 96(8.1) |  |  |  |
| ercc3 (958607) | cross-regional | 6(1.5) | 401(98.5) | 0.004 | 0.948 | 0.002 |
|  | Non-cross-regional | 17(1.4) | 1172(98.6) |  |  |  |
| ercc3 (959167) | cross-regional | 0(0.0) | 407(100.0) | 3.917 | 0.048 | -0.057 |
|  | Non-cross-regional | 15(1.3) | 1174(98.7) |  |  |  |
| pepD (1100234) | cross-regional | 407(100.0) | 0(0.0) | - | - | -. |
|  | Non-cross-regional | 1189(100.0) | 0(0.0) |  |  |  |
| fadH (1306259) | cross-regional | 387(95.1) | 20(4.9) | 4.905 | 0.027 | 0.055 |
|  | Non-cross-regional | 1091(91.8) | 98(8.2) |  |  |  |
| fadH(1306322) | cross-regional | 0(0.0) | 407(100.0) | 7.284 | 0.007 | -0.068 |
|  | Non-cross-regional | 21(1.8) | 1168(98.2) |  |  |  |

**Supplement Table4**(Continue)

| **Gene (position)** | **Category** | **Mutation** | **No mutation** | **Chi-square** | ***P* value** | **COR** |
| --- | --- | --- | --- | --- | --- | --- |
| fadH(1307598) | cross-regional | 407(100.0) | 0(0.0) | - | 1.000 | 0.021 |
|  | Non-cross-regional | 1187(99.8) | 2(0.2) |  |  |  |
| ogt(1477346) | cross-regional | 3(0.7) | 404(99.3) | 2.437 | 0.119 | -0.039 |
|  | Non-cross-regional | 22(1.9) | 1167(98.1) |  |  |  |
| ogt(1477522) | cross-regional | 52(12.8) | 355(87.2) | 15.660 | 7.581e-5 | 0.099 |
|  | Non-cross-regional | 78(6.6) | 1111(93.4) |  |  |  |
| ogt(1477596) | cross-regional | 274(67.3) | 133(32.7) | 6.777 | 0.009 | -0.065 |
|  | Non-cross-regional | 880(0.74) | 309(26.0) |  |  |  |
| lipI(1576481) | cross-regional | 351(86.2) | 56(13.8) | 0.843 | 0.361 | -0.023 |
|  | Non-cross-regional | 1046(88.0) | 143(12.0) |  |  |  |
| lipI(1576527) | cross-regional | 352(86.5) | 55(13.5) | 0.777 | 0.380 | -0.022 |
|  | Non-cross-regional | 1048(88.1) | 141(11.9) |  |  |  |
| tkt(1630148) | cross-regional | 407(100.0) | 0(0.0) | - | - | -. |
|  | Non-cross-regional | 1189(100.0) | 0(0.0) |  |  |  |
| inhA(1674210) | cross-regional | 10(2.5) | 397(97.5) | 0.822 | 0.365 | -0.023 |
|  | Non-cross-regional | 40(3.4) | 1149(96.6) |  |  |  |
| lgt(1814428) | cross-regional | 8(20.0) | 399(98.0) | 1.058 | 0.304 | 0.026 |
|  | Non-cross-regional | 15(1.3) | 1174(98.7) |  |  |  |
| rpsA(1834177) | cross-regional | 382(93.9) | 25(6.1) | 3.785 | 0.052 | 0.049 |
|  | Non-cross-regional | 1079(90.7) | 110(9.3) |  |  |  |
| rpsA(1834776) | cross-regional | 0(0.0) | 407(100.0) | 10.111 | 0.001 | -0.080 |
|  | Non-cross-regional | 29(2.4) | 1160(97.6) |  |  |  |
| tlyA(1917972) | cross-regional | 407(100.0) | 0(0.0) | - | 1.000 | 0.021 |
|  | Non-cross-regional | 1187(99.8) | 2(0.2) |  |  |  |
| lipJ(2147022) | cross-regional | 407(100.0) | 0(0.0) | 0.357 | 0.550 | 0.029 |
|  | Non-cross-regional | 1185(99.7) | 4(0.3) |  |  |  |
| helZ(2361257) | cross-regional | 0(0.0) | 407(100.0) | 11.178 | 0.001 | -0.084 |
|  | Non-cross-regional | 32(2.7) | 1157(97.3) |  |  |  |
| helZ(2361311) | cross-regional | 8(20.0) | 399(98.0) | 4.091 | 0.043 | -0.051 |
|  | Non-cross-regional | 49(4.1) | 1140(95.9) |  |  |  |
| helZ(2361604) | cross-regional | 405(99.5) | 2(0.5) | 0.000 | 1.000 | -0.011 |
|  | Non-cross-regional | 1185(99.7) | 4(0.3) |  |  |  |
| helZ(2362041) | cross-regional | 407(100.0) | 0(0.0) | - | - | -. |
|  | Non-cross-regional | 1189(100.0) | 0(0.0) |  |  |  |
| fadD15 (2448458) | cross-regional | 383(94.1) | 24(5.9) | 3.873 | 0.049 | 0.049 |
|  | Non-cross-regional | 407(100.0) | 107(90.0) |  |  |  |
| fadD15 (2449629) | cross-regional | 0(0.0) | 407(100.0) | 16.213 | 5.660e-5 | -0.101 |
|  | Non-cross-regional | 46(3.9) | 1143(96.1) |  |  |  |
| acpS (2839689) | cross-regional | 12(2.9) | 395(97.1) | 5.373 | 0.020 | -0.058 |
|  | Non-cross-regional | 70(5.9) | 1119(94.1) |  |  |  |

**Supplement Table4**(Continue)

| **Gene (position)** | **Category** | **Mutation** | **No mutation** | **Chi-square** | ***P* value** | **COR** |
| --- | --- | --- | --- | --- | --- | --- |
| fas (2841022) | cross-regional | 380(93.4) | 27(6.6) | 2.802 | 0.094 | 0.042 |
|  | Non-cross-regional | 1078(90.7) | 111(9.3) |  |  |  |
| fas (2847281) | cross-regional | 383(94.1) | 24(5.9) | 4.058 | 0.044 | 0.050 |
|  | Non-cross-regional | 1081(90.9) | 108(9.1) |  |  |  |
| relA (2908252) | cross-regional | 2(0.5) | 405(99.5) | 16.487 | 4.899e-5 | -0.102 |
|  | Non-cross-regional | 59(50) | 1130(95.0) |  |  |  |
| arsA (3001498) | cross-regional | 17(4.2) | 390(95.8) | 26.357 | 2.838e-7 | 0.129 |
|  | Non-cross-regional | 7(0.6) | 1182(99.4) |  |  |  |
| mtr (3165636) | cross-regional | 283(69.5) | 124(30.5) | 6.902 | 0.009 | -0.066 |
|  | Non-cross-regional | 905(76.1) | 284(23.9) |  |  |  |
| tesA (3242617) | cross-regional | 11(2.7) | 396(97.3) | 1.353 | 0.245 | 0.029 |
|  | Non-cross-regional | 21(1.8) | 1168(98.2) |  |  |  |
| gatB (3367765) | cross-regional | 407(100) | 0(0) | - | 1.000 | 0.015 |
|  | Non-cross-regional | 1188(99.9) | 1(0.1) |  |  |  |
| cstA (3428183) | cross-regional | 15(3.7) | 392(96.3) | 1.254 | 0.263 | -0.028 |
|  | Non-cross-regional | 407(100) | 1129(95.0) |  |  |  |
| cstA (3428917) | cross-regional | 407(100) | 0(0) | 0.357 | 0.550 | 0.029 |
|  | Non-cross-regional | 1185(99.7) | 4(0.3) |  |  |  |
| agpS (3476350) | cross-regional | 30(7.4) | 377(92.6) | 27.914 | 1.268e-7 | 0.132 |
|  | Non-cross-regional | 407(100) | 1166(98.1) |  |  |  |
| nudC (3571828) | cross-regional | 352(86.5) | 55(13.5) | 0.546 | 0.460 | -0.019 |
|  | Non-cross-regional | 1045(87.9) | 144(12.1) |  |  |  |
| sdhD (3704596) | cross-regional | 407(100) | 0(0) | - | - | -. |
|  | Non-cross-regional | 1189(100) | 0(0) |  |  |  |
| sdhD (3704686) | cross-regional | 12(2.9) | 395(97.1) | 0.733 | 0.392 | -0.021 |
|  | Non-cross-regional | 407(100) | 1143(96.1) |  |  |  |
| sdhD (3704770) | cross-regional | 383(94.1) | 24(5.9) | 4.631 | 0.031 | 0.054 |
|  | Non-cross-regional | 1078(90.7) | 111(9.3) |  |  |  |
| nagA (3719723) | cross-regional | 2(0.5) | 405(99.5) | 1.054 | 0.305 | -0.033 |
|  | Non-cross-regional | 15(1.3) | 1174(98.7) |  |  |  |
| lipF (3906311) | cross-regional | 381(93.6) | 26(6.4) | 3.357 | 0.067 | 0.046 |
|  | Non-cross-regional | 1078(90.7) | 111(9.3) |  |  |  |
| acs (4108495) | cross-regional | 4(10) | 403(99) | 3.175 | 0.075 | -0.045 |
|  | Non-cross-regional | 29(2.4) | 1160(97.6) |  |  |  |
| acs (4109342) | cross-regional | 12(2.9) | 395(97.1) | 2.692 | 0.101 | -0.041 |
|  | Non-cross-regional | 58(4.9) | 1131(95.1) |  |  |  |
| crp (4116610) | cross-regional | 340(83.5) | 67(16.5) | 0.130 | 0.718 | 0.009 |
|  | Non-cross-regional | 984(82.8) | 205(17.2) |  |  |  |
| crp (4116773) | cross-regional | 12(2.9) | 395(97.1) | 0.027 | 0.869 | -0.004 |
|  | Non-cross-regional | 37(3.1) | 1152(96.9) |  |  |  |

**Supplement Table4**(Continue)

| **Gene (position)** | **Category** | **Mutation** | **No mutation** | **Chi-square** | ***P* value** | **COR** |
| --- | --- | --- | --- | --- | --- | --- |
| pcnA (4392373) | cross-regional | 8(20.0) | 399(98.0) | 1.777 | 0.183 | 0.033 |
|  | Non-cross-regional | 13(1.1) | 1176(98.9) |  |  |  |

COR, correlation coefficient.

-means there is no result in statistical software or the result was too large and nonsense.
